# A Structure-Based Drug Discovery Paradigm

**DOI:** 10.3390/ijms20112783

**Published:** 2019-06-06

**Authors:** Maria Batool, Bilal Ahmad, Sangdun Choi

**Affiliations:** Department of Molecular Science and Technology, Ajou University, Suwon 16499, Korea; mariabatool.28@gmail.com (M.B.); bilalpharma77@gmail.com (B.A.)

**Keywords:** deep learning, artificial intelligence, neural network, structure-based drug discovery, virtual screening, scoring function

## Abstract

Structure-based drug design is becoming an essential tool for faster and more cost-efficient lead discovery relative to the traditional method. Genomic, proteomic, and structural studies have provided hundreds of new targets and opportunities for future drug discovery. This situation poses a major problem: the necessity to handle the “big data” generated by combinatorial chemistry. Artificial intelligence (AI) and deep learning play a pivotal role in the analysis and systemization of larger data sets by statistical machine learning methods. Advanced AI-based sophisticated machine learning tools have a significant impact on the drug discovery process including medicinal chemistry. In this review, we focus on the currently available methods and algorithms for structure-based drug design including virtual screening and de novo drug design, with a special emphasis on AI- and deep-learning-based methods used for drug discovery.

## 1. Introduction

In the drug discovery process, the development of novel drugs with potential interactions with therapeutic targets is of central importance. Conventionally, promising-lead identification is achieved by experimental high-throughput screening (HTS), but it is time consuming and expensive [1]. Completion of a typical drug discovery cycle from target identification to an FDA-approved drug takes up to 14 years [2] with the approximate cost of 800 million dollars [3]. Nonetheless, recently, a decrease in the number of new drugs on the market was noted due to failure in different phases of clinical trials [4]. In November 2018, a study was conducted to estimate the total cost of pivotal trials for the development of novel FDA-approved drugs. The median cost of efficacy trials for 59 new drugs approved by the FDA in the 2015–2016 period was $19 million [5]. Thus, it is important to overcome limitations of the conventional drug discovery methods with efficient, low-cost, and broad-spectrum computational alternatives.

**Figure 1:**
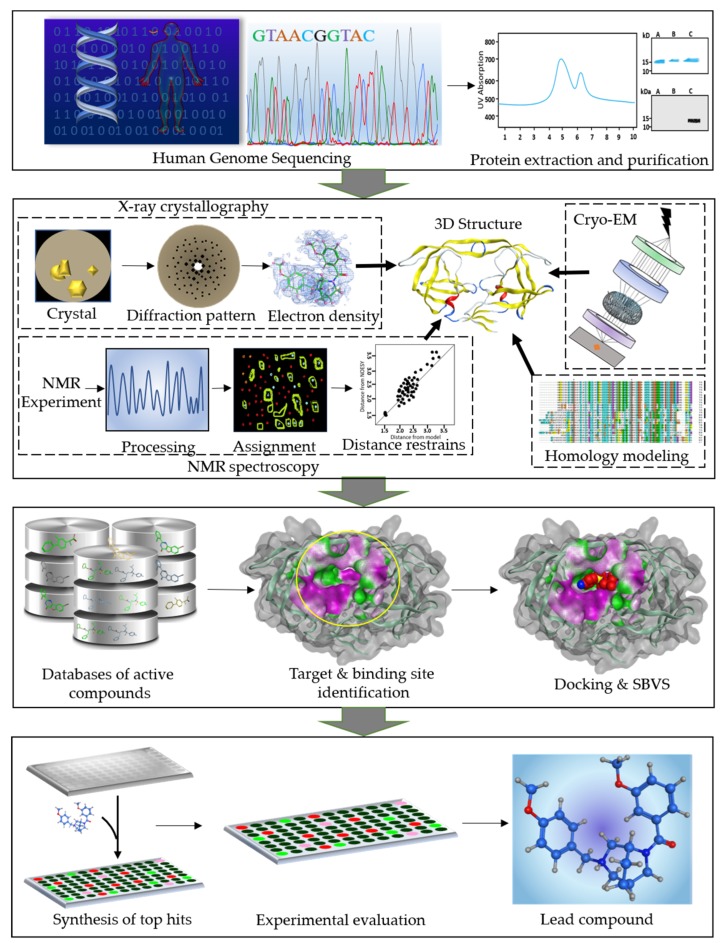
A workflow diagram of structure-based drug design (SBDD) process. The first panel shows the human genome sequencing followed by extraction and purification of the target proteins. Second panel represents the structure determination of the therapeutically important proteins using integrative structural biology approaches. Third panel represents the database preparation of the active compounds. The next step is identification of the druggable target protein and its binding site. Subsequently, the databases of active compounds are screened and docked into the binding cavity of the target protein. In the last panel, the identification of the potent lead compound is shown. The top hit compounds obtained as a result of virtual screening and docking are synthesized and tested in vitro. Further modifications can be done for optimization of the lead compound.

In contrast to the traditional drug discovery method (classical or forward pharmacology), rational drug design is efficient and economical. The rational drug design method is also known as reverse pharmacology because the first step is to identify promising target proteins, which are then used for screening of small-molecule libraries [6]. Striking progresses have been made in structural and molecular biology along with advances in biomolecular spectroscopic structure determination methods. These methods have provided three-dimensional (3D) structures of more than 100,000 proteins [7]. In conjunction with the storage of (and organizing) such data, there has been much hype about the development of sophisticated and robust computational techniques. Completion of the Human Genome Project and advances in bioinformatics increased the pace of drug development because of the availability of a huge number of target proteins. The availability of 3D structures of therapeutically important proteins favors identification of binding cavities and has laid the foundation for structure-based drug design (SBDD). This is becoming a fundamental part of industrial drug discovery projects and of academic researches [8]. SBDD is a more specific, efficient, and rapid process for lead discovery and optimization (Figure 1) because it deals with the 3D structure of a target protein and knowledge about the disease at the molecular level [9]. Among the relevant computational techniques, structure-based virtual screening (SBVS), molecular docking, and molecular dynamics (MD) simulations are the most common methods used in SBDD. These methods have numerous applications in the analysis of binding energetics, ligand–protein interactions, and evaluation of the conformational changes occurring during the docking process [10]. In recent years, developments in the software industry have been driven by a massive surge in software packages for efficient drug discovery processes. Nonetheless, it is important to choose outstanding packages for an efficient SBDD process [11]. Briefly, automation of all the steps in an SBDD process has shortened the SBDD timeline [8]. Moreover, the availability of supercomputers, computer clusters, and cloud computing has sped up lead identification and evaluation. In this review, we offer an overview of the SBDD process and the methods being used in the present era. Moreover, we provide an in-depth discussion about the machine learning (ML) methods intended to speed up this process and big-data handling.

## 2. An Overview of SBDD Process

In the entire drug discovery paradigm, SBDD is the most powerful and efficient process. Computational resources serve as an efficient technology for accelerating the drug discovery process, which includes various screening procedures, combinatorial chemistry, and calculations of such properties as absorption, distribution, metabolism, excretion and toxicity (ADMET) [12]. SBDD is an iterative process and it proceeds through multiple cycles leading an optimized drug candidate to clinical trials. Generally, a drug discovery process consists of four steps: the discovery phase, development phase, clinical trial phase, and registry phase. In the first phase, a potential therapeutic target and active ligands are identified. The fundamental step involves cloning of the target gene followed by the extraction, purification, and 3D structure determination of the protein. Many computer algorithms can be used to dock the huge databases of small molecules or fragments of compounds into the binding cavity of the target protein. These molecules are ranked according to a scoring system based on electrostatic and steric interactions with the binding site. Thorough investigation of electrostatic properties of the binding site, including the presence of cavities, clefts, and allosteric pockets can be carried out using a 3D structure of the target molecule. Current SBDD methods consider the key features of the binding cavity of the therapeutic target to design efficient ligands [13,14]. In the second phase, the top hits are synthesized and optimized [15]. Furthermore, the top-ranked compounds with high affinity for selective modulation of the target protein are tested in vitro in biochemical assays. These ligands interfere with crucial cellular pathways, thereby leading to the development of drugs with a desired therapeutic and pharmacological effect [16]. Biological properties like efficacy, affinity, and potency of the selected compounds are evaluated by experimental methods [17]. The next step is to determine the 3D structure of the target protein in complex with the promising ligand obtained in the first phase. The 3D structure provides detailed information about the intermolecular features that aid in the process of molecular recognition and binding of the ligand. Structural insights into the ligand–protein complex help with the analysis of various binding conformations, identification of unknown binding pockets, and ligand–protein interactions; elucidation of conformational changes resulting from ligand binding; and detailed mechanistic studies [7]. Subsequently, multiple iterations increase the efficacy and specificity of the lead. The third phase includes clinical trials of the lead compounds. Those compounds that pass the clinical trials proceed to the fourth phase in which the drug is distributed in the market for clinical use.

**Figure 2:**
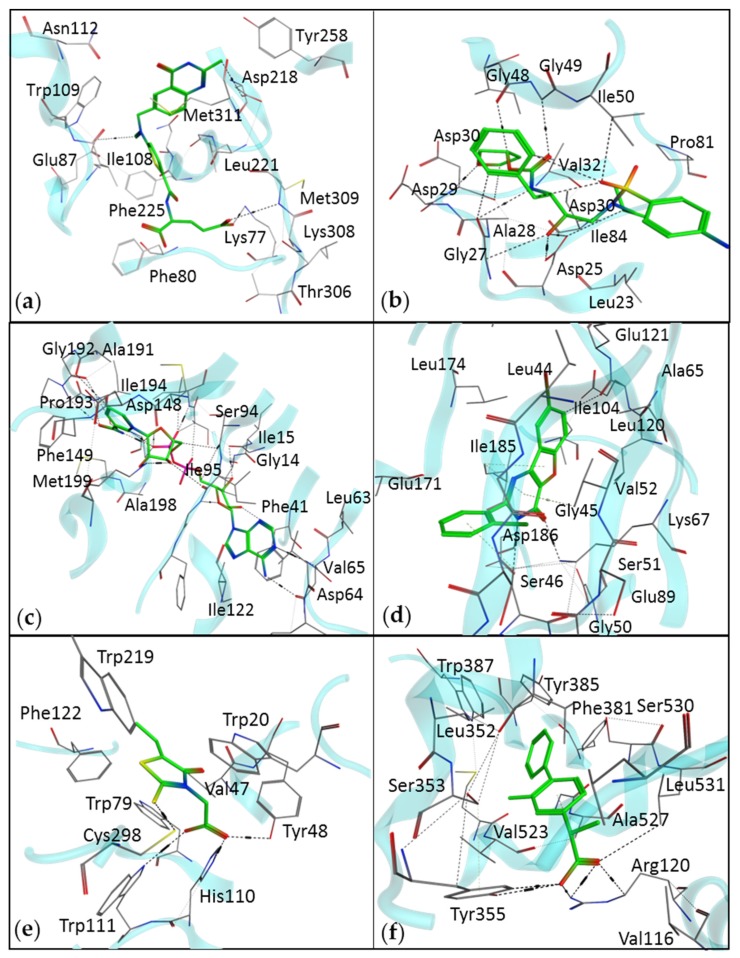
The interaction diagram of drugs identified by SBDD methods, with their respective therapeutic targets. (**a**) An interaction of raltitrexed with thymidylate synthase (Protein Data Bank (PDB) ID: 5X5Q). (**b**) An interaction of amprenavir with HIV protease (PDB ID: 3EKV). (**c**) Isoniazid, a drug for tuberculosis, identified by the SBVS method (PDB ID: 1ENY). (**d**) Pim-1 kinase inhibitor, benzofuropyrimidine, for the treatment of various types of cancers (PDB ID: 4ALU). (**e**) Epalrestat is an aldose reductase inhibitor (PDB ID: 4JIR). (**f**) Flurbiprofen is a cyclooxygenase 2 inhibitor (PDB ID: 3PGH).

SBDD is a computational technique widely used by pharmaceutical companies and scientists. There are numerous drugs available on the market that have been identified by SBDD. Human immunodeficiency virus (HIV)-1-inhibiting FDA-approved drugs represent the foremost success story of SBDD [18]. Moreover, other drugs identified by the SBDD technique include a thymidylate synthase inhibitor, raltitrexed [8]; amprenavir, a potential inhibitor of HIV protease discovered by protein modeling and MD simulation [18,19]; and the antibiotic norfloxacin [20]. Other examples of success cases of drug discovery via SBDD methods are listed in Table 1, whereas the interactions of these drugs with respective targets are shown in Figure 2. Some of the failure cases have also been documented; for example, RPX00023 has been reported as an antidepressant that was claimed to have an agonistic activity toward receptor 5-HT1A, but it inhibited the receptor [21]. These failure cases are the reason for limitations in SBDD strategies. Although SBDD workflow includes various efficient methods, they all have certain restrictions, which require further research work.

### 2.1. Target Protein and Binding Site Identification

The basic step in a typical SBDD process is target protein identification and validation [29]. The 3D structures of all therapeutically important proteins are determined experimentally by integrative structure biology techniques such as: NMR, X-ray crystallography, or cryo-electron microscopy but if a solution structure is not available, in silico methods are used to model the protein’s 3D structure. There are three well-known structure prediction methods such as comparative modeling, threading, and ab initio modeling. Among them, homology modeling is one of the best and reliable approaches because it predicts the 3D structure of a target protein on the basis of the knowledge about the structure of homologous proteins with >40% similarity [2]. Once the 3D structure of the target is predicted, it is necessary to validate the model by checking the stereochemical properties in a Ramachandran plot. It shows the possible conformations of ψ and φ angles for all amino acid residues present in the protein structure [30]. There are many other methods for validation of the model [2,31,32].

After the structure of the target protein is resolved, the next step is to identify the binding pocket. This is a small cavity where ligands bind to the target to produce the desired effect. Therefore, it is necessary to identify the appropriate site on the target protein. In spite of the protein’s dynamic nature, there are a few methods capable of spotting the potential binding residues. These methods consider the knowledge about interaction energy and van der Waals (vdW) forces for binding site mapping. Many methods have been developed for binding site mapping by interaction energy calculations specifically for SBDD. This method identifies particular sites on the target protein which interact favorably with important functional groups on drug-like molecules [33]. These methods identify energetically favorable interactions of specific probes with the proteins. Q-SiteFinder [33] is an energy-based method commonly used for binding site prediction. This method calculates vdW interaction energies of proteins with a methyl probe. Those with favorable energies are retained and clustered. These probe clusters are ranked based on their total interaction energies. In addition, interacting protein residues are functionally annotated to determine the binding site. The next step is hit discovery, which is done by docking of compound libraries into the binding cavity of the target protein. In the initial phases of lead discovery, it is important to choose a specific set of ligands that play a key part in the lead identification and optimization [34]. For hit hunting, SBDD integrates two divergent methods (i.e., virtual screening (VS) and de novo design).

### 2.2. Virtual Screening: A Lead Identification Approach

In medicinal chemistry, VS is a robust approach to lead identification [3]. In VS, databases of millions of drug-like or lead-like compounds are screened computationally against the target proteins with well-known 3D structures. The screening of compound libraries is accomplished by docking, where ligands are filtered based on their binding affinity [35,36]. The top hits of the computational screening are then tested in vitro [3,37]. VS is classified into two major types: ligand-based VS (LBVS) and SBVS. In LBVS, biological data are analyzed to separate inactive compounds from the active compounds. This information is then employed to identify highly active scaffolds on the basis of consensus pharmacophores [38], similarity, or various descriptors. In SBVS, the knowledge about the 3D structure of the target protein is necessary. The target protein is docked with the huge libraries of drug-like compounds, available commercially, via computer algorithms. A scoring function is executed to evaluate the binding force of the docked complex followed by experimental assays to validate the binding. The scoring of ligands is a critical step in SBVS. Unlike ligand-based methods, structure-based approaches do not rely on already available experimental data.

### 2.3. De Novo Drug Design

De novo drug design is a method of building novel chemical compounds starting from molecular units. The gist of this approach is to develop chemical structures of the small molecules that bind to the target binding cavity with good affinity [39]. Generally, a stochastic approach is used for de novo design, and it is important to take the search space knowledge into consideration in the design algorithm. The two designs, positive and negative, are being used. In the former design, a search is restricted to the specific regions of chemical space with higher probability of finding hits having required features. In contrast, the search criteria are predefined in the negative mode, to prevent the selection of false positives [40]. The chemical compound designing by computational techniques can be related to imitation of synthetic chemistry, while scoring functions perform binding assays [41]. Critical assessment of candidates is crucial for the design process, and the scoring function is one of the assessment tools. Multiple scoring functions can be employed parallelly for multi-objective drug design [42], which considers multiple features at once.

Two methods—(i) ligand-based and (ii) receptor-based de novo drug design—can be used. The latter approach is more prevalent. The quality of target protein structures and accurate knowledge about its binding site are important for receptor-based design because suitable small molecules are designed by fitting the fragments into the binding cavities of the receptors. This could be either done by means of a computational program or by cocrystallization of the ligand with the receptor [43]. There are two techniques for receptor-based design: building blocks, either atoms or fragments such as single rings, amines, and hydrocarbons are linked together to form a complete chemical compound or simply by growing a ligand from a single unit. In the fragment-linking method, the binding site is identified to map the probable interacting points for different functional groups present in the fragments [44]. These functional groups are attached together to build an absolute compound. In the fragment-growing technique, the growth of fragments is accomplished within the binding site monitored by suitable search algorithms [45]. These search algorithms involve scoring functions to assess the probability of growth. Fragment-based de novo design uses the whole chemical space to generate novel compounds. In case of the linking approach, the selection of linkers is critical. Fragment anchoring in the binding site can be performed by (i) the outside-in approach and (ii) the inside-out approach. In the former approach, the building blocks are primarily arranged at the periphery of the binding site, and it grows inward. In the course of the inside-out approach, building blocks are casually fitted into the binding site and built outward [10].

### 2.4. Molecular Docking

Docking is a technique of virtual simulation of molecular interactions [46]. Molecular docking predicts the conformation and binding of ligands within a target active site with high accuracy; therefore, it is the most popular technique in SBDD [47,48]. This method can be applied to study important molecular phenomena such as a ligand-binding pose and intermolecular interactions for stability of a complex [49]. Moreover, docking algorithms predict binding energies and rank the ligands by means of various scoring functions [49,50]. The appropriate ligand-binding conformation depends on two factors: (i) large conformational space defining possible binding poses and (ii) explicit prediction of binding energy correlating with each conformation [51]. Multiple iterations are performed, until the minimum energy state is attained, in which ligand-binding is assessed by various scoring functions [7].

There are two types of molecular docking: flexible-ligand search docking and flexible-protein docking. In flexible-ligand search docking, three types of algorithms are designed to deal with the ligand flexibility. These algorithms are the stochastic method, systematic method, and simulation method [52]. The systematic algorithms are aimed at analyzing degrees of freedom. This task can be accomplished by the fragmentation method, one of the frequently used techniques. In this method, a ligand grows gradually inward in a binding cavity [52,53]. In the conformational search technique, rotatable bonds of the molecule are rotated 360° systematically at a fixed-increment rate, or in the database approach, pregenerated libraries of conformational ensembles are utilized for ligand flexibility. In the stochastic algorithms, random modifications are applied to a single ligand or a group. These modifications are accepted or rejected depending upon probability functions such as genetic algorithm methods [52,54] and the Monte Carlo (MC) method. Lastly, MD simulation is a comprehensive technique for studying the dynamic behavior of macromolecules. Energy minimization is implemented as integration with simulations to achieve local minima. The algorithms available for energy minimization are the Newton–Raphson method, steepest descent, least squares methods, and a conjugate gradient [52]. Many biological systems show movements upon ligand binding; thus, in the flexible-protein docking method, the receptor remains flexible during the docking procedure to mimic the natural biological environment. In addition to the full protein movement, in a few cases, small motions are also noticed such as side chain rearrangement or movement of highly flexible loops. MD and MC methods are suitable for flexible-protein docking [55,56].

### 2.5. Scoring Functions

A scoring function helps a docking program to delve into the ligand-binding site. Once a significant binding conformation is identified, the scoring function calculates binding affinity. Accordingly, scoring functions are thought to have a substantial impact on docking. Scoring functions are trained on a training dataset of a similar class of compounds for which their experimental binding affinity is available. Scoring functions are divided into four general classes: force field, empirical, knowledge-based, and machine learning (ML) [57,58,59]. The force field is calculated by estimating the intermolecular interactions such as electrostatic and vdW forces between the binding partners. Empirical scoring functions are calculated based on the atom numbers in the ligand and target protein and are used for affinity and pose prediction [60]. The latter includes hydrophobic forces, hydrophilic forces, hydrogen bonding, and entropy. A statistical method called multiple linear regression is employed to fit scoring-function coefficients. A knowledge-based scoring function depends on statistical potentials of intermolecular interactions. This method is based purely on the assumption that frequently occurring functional groups or a certain type of atoms are energetically favorable and contribute to binding affinity [61]. In contrast to classical scoring functions, ML methods do not constrain analysis to a predefined functional form among structural features and binding affinity values [62]. ML methods are dynamic techniques for construction and optimization of models to predict a binding pose and affinity. Lately, the development of novel scoring functions by ML is becoming popular [63]. These methods implicitly take into consideration the interactions between a ligand and target while ignoring error-prone interactions. Furthermore, different methods of the ML technique such as random forest (RF), support vector machine (SVM), and neural networks (NN) work with nonlinear dependence among binding interactions. Thus, ML-based scoring functions perform better than others do in case of binding energy calculations [1]. Another scoring function known as consensus scoring employs collective scores to minimize the error rate in individual scores and to increase the possibility of true positive selection [52].

The efficiency of various scoring functions has been compared in many studies [64,65,66,67,68], regarding binding affinity prediction, reproducibility of a known binding conformation, and ranking of a library. All modern scoring functions have different accuracy rates under different conditions. Thus, none of the scoring functions can outperform the others. However, consensus scoring function can perform better than single-scoring approach and is widely used in various bioinformatics applications. Consensus scoring function compensates the limitations of single-scoring functions. It improves the hit rate by combining multiple scoring functions based on a simple cause: the true value tends to be closer to the mean value of replicated experiments [69]. In case of single-scoring functions, a binding pose can be predicted accurately, but in terms of binding energy calculations, there is still a need to improve the performance of current scoring functions. Hence, a lot of efforts have been made to upgrade the abilities of the currently available scoring functions. Prevalent methods include the addition of certain features for calculation of entropic and solvation effects [70], development of a consensus scoring function to overcome the limitations of others [69], and calculations of quantum-energy terms [71]. Targeted scoring functions are known to significantly enhance VS performance and might be a solution to the limitations of other scoring functions [72]. Such scoring functions generate output with higher probability of true hits and a decreased rate of false positives.

## 3. Big Data in Drug Discovery

The “big data” approach influences our daily life, and drug discovery is not an exception. By current computational techniques, molecular characteristics can be studied in a logical and systematic manner. The data collected from each compound can be subjected to analyses from different perspectives [73]. In the modern era of technology, there has probably been an increase in the size of data generation. According to a recent estimate, the total size of stored data is approximately two zettabytes (10^21^) with expected doubling every two years [74]. Hence, excavation of massively produced digital information offers a multitude of opportunities to increase productivity. Nevertheless, apart from the volume and production rate of big data, the variety and complexity of big data pose challenges for effective analysis [75]. Furthermore, sometimes generated data contain inconsistencies, such as missing or incomplete information, errors, and duplications, thereby affecting the outcomes of accurate simulation and analytical activities. Therefore, preliminary analysis and curation are required as advanced measures to ensure fairness, accuracy, and experimental efficacy [76]. On the other hand, precollection and curation measures vary among research communities, depending on preceding observations and experimental records. Yet, there is high demand for a simple, unified, and well-established curation protocol that ensures the quality of generated simulation and analytical datasets.

Several studies examined the impact of quality on research activities [77]. Several others recommend conducting a fair evaluation of the quality and impact of a particular work [78]. Hence, the existing standard of research continues to adhere to the “less-is-more” principle. Big data have played a vital role in medicinal and combinatorial chemistry, whereas HTS contributes to the generation of a huge amount of data over a short span of time. Big data dependency will likely increase as the perception of personalized medicine improves. Earlier, big data have been regarded as the beginning of computation-oriented medicinal chemistry (i.e., processing stacks of generated data, resulting in shortening of the time taken to complete a drug development cycle). For instance, a well-known global pandemic spanning more than 40 years, HIV, has infected more than 37 million people, where only 57% are being treated with antiviral agents (World Health Organization (WHO), 2018). In the past few years, many studies have addressed the inhibition of viral reverse transcriptase and/or integrase [79,80]. Although this technique has proven effective enough, it comes with several shortcomings such as viral resistance and poor bioavailability.

In the early 1990s, the roles of chemokines and CD4^+^ cells were described. Chemokine activity is associated with their G-protein-coupled receptors (GPCRs); in the CCR5 case, it is a “C-C” receptor with 75% homology to CCR2 [81]. With the emergence of CCR5 as an interesting and a druggable novel target to combat HIV, numerous pharmaceutical firms turned to their GPCR inhibitor libraries in search of a putative ligand for this protein. A strong lead, an imidazopyridine (UK107543) was identified by Pfizer, a well-known pharmaceutical company, using HTS [82]. Maraviroc (Selzentry), an antiretroviral drug, classified as an entry inhibitor was later declared as an approved drug for HIV-1 treatment by the FDA [83]. Such real-world use cases spotlight the significance of big data resources in medicinal chemistry. Therefore, among medicinal chemists, we are seeing a major demand for rational awareness of data-driven processes and for information-handling skills [84].

From this standpoint, the scientific communities started investing in the development of applications, tools, and software to handle massively generated and already stored data. Nevertheless, a major concern limiting the usability of these computational platforms includes security and privacy concerns for the users [85]. Aside from these factors, freely and publicly accessible resources provide a versatile collection, which can be manipulated beyond the pharmaceutical scope [86].

## 4. Artificial Intelligence and Machine Learning in Drug Discovery

Artificial intelligence (AI) mimics human behavior by simulating human intelligence by computer techniques [87]. ML, a subfield of AI, uses statistical methods for learning with or without being programmed [88]. In the drug development process, AI has shifted the mood from hype to hope [87]. Computational technologies and ML algorithms have revolutionized drug discovery in the pharmaceutical industry. Integration of ML algorithms in an automatic manner–to discover new compounds by analyzing, learning, and explaining pharmaceutical big data–is the application of AI to drug design [89]. Big Pharma is increasing investment in AI; this situation shows the truth behind the use of ML algorithms to identify and screen potential drug candidates. For instance, SYNSIGHT has introduced an AI-based integrated platform in combination with VS and molecular modeling to create huge biological models for drug development [90]. Many leading biopharmaceutical companies are collaborating to integrate AI and ML methods with their drug discovery pipelines. Pfizer has been collaborating with IBM since December 2016 to take advantage of their multicloud platform Watson [91] for immuno-oncology drug discovery [92]. Similarly, Exscientia Ltd., a UK-based world class AI-driven drug design company [93] is collaborating with Sanofi to find a cure for metabolic disorders [94], and Clegene, another leading pharmaceutical company, aims to accelerate drug discovery in the areas of autoimmunity and oncology [95]. Recently, Exscientia announced a success story in collaboration with GlaxoSmithKline (GSK), where they claimed the discovery of a highly potent lead molecule for the treatment of chronic obstructive pulmonary disease by means of AI-based drug discovery workflow [96].

ML success has been repeatedly demonstrated in classification, generative modeling, and reinforcement learning (RL). Different categories of ML are supervised learning, unsupervised learning, and RL. The subcategory of supervised learning, classification, and regression methods predicts the model on the basis of input and output data sources. Supervised ML is applicable to a disease in diagnostic methods, ADMET in a classification method’s output, and to drug efficacy in regression methods [97]. SVMs with supervised ML algorithms use binary activity prediction to distinguish between a drug and nondrug [98,99] or between specific and nonspecific compounds [100,101]. SVM classification is performed in LBVS to rank the database compounds by decreasing activity probability. To minimize error in SVM ranking, optimized special ranking functions are used [101]. The clustering method for an unsupervised learning category can discover a disease subtype as outputs, while a feature-finding method can identify a target in a disease [102,103]. Decision-making RL maximizes its performance in de novo drug design via modeling and quantum chemistry. RL is less dependent on dataset learning. With RL, the desired physical and biological properties of newly generated chemical structures can be biased [104]. ML exploits the relationship between a biological activity and chemical structure during drug design. Structure prediction of biological targets (protein structure, binding pocket, transmembrane regions, and phosphorylation and glycosylation sites) and quantitative structure–activity relationship (QSAR) models, pharmacophore models, molecular docking analysis, and ranking/scoring functions in similarity searches–can be implemented and statistically validated by ML techniques [105]. Classifying a pharmacokinetic and toxicological (ADMET) profile, discovery or optimization of biologically active hit compounds, and the constructed model or biological activity of a new ligand can aid with a drug discovery process at several steps by ML techniques [106]. Multiple ML models can be used to drive multiparameter optimization. The output of ML methods depends on multiple parameters like diversity of the training dataset, an ability to handle imbalanced datasets of active and inactive compounds in the library and defining precise parameters to cover full chemical space including active and inactive molecules [107]. Proficient ML models can be developed to screen huge libraries which generate few false positives and a good number of active compounds in the output. This goal can be attained using versatile training datasets comprising predicted inactive compounds [108,109].

## 5. The Role of Deep Learning in Drug Design

**Figure 3:**
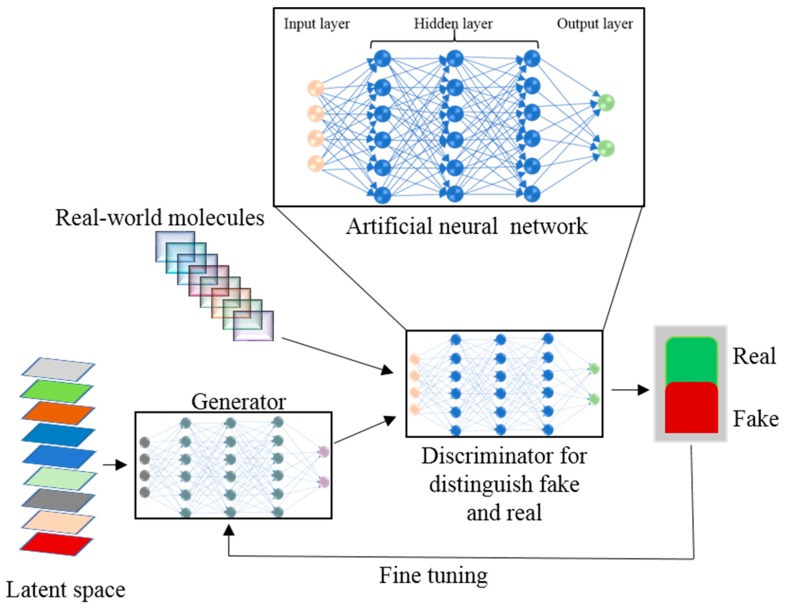
A workflow of the generative adversarial network approach with an artificial neural networks (ANN) for new molecule design.

NN represent a supervised neurology-inspired ML technique that is employed routinely and successfully to address such issues as speech and image recognition. Artificial neural networks (ANNs) are ML algorithms that operate as neurons in the brain: they receive numerous input signals and generate an activation response by calculating a weighted sum of the inputs through a nonlinear activation function and pass the output signal to subsequent connected neurons [110]. The basic structure of an ANN consists of an input layer, hidden layer, and the output layer (Figure 3).

In the ANN, the processing nodes are either fully or partially connected. From input nodes, the input variables are taken and are transformed through hidden nodes into the output nodes where output values are calculated. By back-propagation methods, the ANN training is done in an iterative fashion to train the network [111]. Due to overfitting, a diminishing gradient, and other problems, the traditional ANN methods have not performed well and have been replaced by other ML algorithms like RF [112] and SVM [113]. The deep learning (DL) concept has originated from ANN’s feedforward NNs with many hidden layers [114]. DL’s recent development has given the ANN a renaissance. DL is changing our everyday life and has achieved huge success in self-driving cars, computer games, speech recognition, natural language processing, and other applications [115]. With the rapid explosion of chemical “big data” from combinatorial synthesis and HTS, ML techniques have become an indispensable tool for drug designers to retrieve chemical information from large compound databases to design drugs rationally. Big data volume, velocity, variety, and veracity characterization are not possible via traditional QSAR approaches. ML techniques are more efficient than the physical model for scaling big datasets. DL, being the data-hungry ML algorithm for analyzing and exploring big data, is in high demand. As compared to other ML methods, the DL architecture is flexible [116]. Atomwise, the first DL-based technology for structure-based small-molecule drug discovery has helped to design new potential drugs for 27 disease targets with accuracy and precision [117]. A straightforward method with a fully connected deep neural network (DNN) is used for model building of compounds having the same number of molecular descriptors. To the Merck Kaggle challenge dataset, Dahl et al. [118] applied a DNN and showed better performance as compared to RF on 13 of the total 15 targets. DNNs can handle thousands of descriptors without overfitting and feature selection problems as in the traditional ANN, in an optimized manner, owing to the number of nodes and hidden layers. Mayr et al.’s multitasking DNN method won the Tox21 dataset challenge consisting of 12,000 compounds for 12 high-throughput toxicity assays. In this challenge the computational toxicity prediction of chemicals and drugs was given. The chemical structures and assay measurements from stress and nuclear receptor signaling pathway assays for 12 different toxic effects were available to the participants to check structure-activity relationships. Mayer et al. developed a DeepTox pipeline for toxicity prediction which uses deep learning algorithms. DeepTox normalizes the chemical structures followed by computation of the chemical descriptors. The computed descriptors are used in DL methods to predict the toxicity of chemicals. Later, these models are combined to ensembles [119]. Statistically, a DNN outperforms other ML models such as SVM [120], RF, and others when applied to seven datasets selected from ChEMBL database [121]. In variational autoencoder (VAE), an encoder NN generates a chemical structure via unsupervised learning to map chemical structures from a database onto a latent space. The trained VAE from the latent vector in the latent space transforms the molecular structure into a simplified molecular-input line-entry system (SMILES) string. Kadurin et al. [122] have generated new structures having specific anticancer properties by coupling the generative adversarial network (GAN) with VAE. In a GAN (Figure 3), two ANN models—the generator and discriminator—are trained simultaneously and generate a new molecule from scratch by optimizing a different and opposing objective function in a zero-sum game [123]. A reinforced adversarial neural computer (RANC) with DL architecture, based on the GAN paradigm and RL, generates unique and adequate structures [124]. The RANC uses the SMILES string dataset with key distribution of chemical features like molecular weight, log P, and topological polar surface area for de novo design of small molecules against different biological targets and pathways. Relevant to drug discovery, RANC trained on SMILES string representation outperforms other methods on several metrics [124]. Segler et al. [125] and Yuan et al. [126] have used a recurrent neural network (RNN) for new structure generation acknowledging its success in natural language processing. RNN generates molecular structures by using the probability distribution learning on the SMILES string training set. Target specific libraries were generated by Segler et al. [125] while exploring the RNNs. RNN together with deep Q-learning the RL technology generates SMILES with desirable properties like quantitative estimate of drug-likeness (QED) [127] and clogP [128]. Olivecrona et al. overcame the incorporation of handwritten rules for undesirable structure penalties by tuning the pretrained RNN using the policy based RL approach [129]. Pereira et al. reported deep-learning-based virtual screening method where they compared 95,316 decoys with 2950 ligands docked on 40 receptors and those ranked by the deep convolutional neural network showed better performance than other docking programs [130]. New molecular fingerprints or focused molecule libraries with modeled pharmacokinetic properties of potential drugs can be generated using DL [131].

## 6. Challenges and Emerging Problems

Drug discovery still faces a lot of challenges, such as (i) upgrading the efficacy of virtual screening methods, (ii) improving computational chemogenomic studies, (iii) boosting the quality and number of computational web sources, (iv) improving the structure of multitarget drugs, (v) enhancing the algorithms for toxicity prediction, and (vi) collaborating with other related fields of study for better lead identification and optimization.

Computer-aided structure-based drug discovery is an integral part of multidisciplinary work. Computer-aided drug discovery can be used in combination with combinatorial chemistry or HTS, by means of various algorithms to prepare combinatorial libraries for HTS, including chemical space characterization [50]. VS is known to shorten the time and cost of HTS methods. The major drawback of VS is that while generating screening libraries, it ignores the protonation and tautomerism effect as well as ionization states of compounds, thereby missing out on significant hits. Availability of limited experimental data and reliable output of computational methods cause researchers to ignore tautomerization, but they are still irresistible [10,132]. In the drug discovery process, ADMET prediction remains a hurdle. Nonetheless, availability of various computational methods for prediction of these values has reduced the time and the number of tests on animals. Further development of informatics toxicology is needed [133].

In the de novo lead generation method, though this process seems to be efficient and acceptable, there are limitations of the linking procedure. The first limitation is that the linking fragments should be placed accurately in the cavity for appropriate linking. Moreover, de novo design is thought to be fully automated, but still there is some work to be done manually, which is quite laborious. Furthermore, compounds designed by this technique are not always easy to synthesize in the laboratory. Thus, new software is needed that considers the synthesis factors while including de novo designing of compounds [10].

In the case of molecular docking, a variety of docking algorithms and scoring functions are available, but it is important to choose an appropriate scoring function, which requires deep knowledge about such software. The limitations of the scoring functions are a major drawback among docking programs because this software provides an efficient evaluation of ligand binding energy but ignores accuracy [52]. Several molecular determinants such as electrostatic interactions and entropy calculations are entirely ignored during ligand-binding energy calculations [48]. No single software package is suitable for work with all types of proteins and ligands. Similarly, accurate binding affinity calculation is still debated [10]. Despite a lot of improvements and currents developments in SBDD, a consistent solution is yet to be developed. To overcome fundamental issues such as considering water molecules and flexibility of a target molecule, revolutionary innovations are still needed.

## Figures and Tables

**Table 1:** The success cases of drug discovery by SBDD methods.

Drug	Drug Target	Target Disease	Technique	Ref.
Raltitrexed	Thymidylate synthase	Human immunodeficiency virus (HIV)	SBDD	[8]
Amprenavir	Antiretroviral protease	HIV	Protein modeling and molecular dynamics (MD)	[18,19]
Isoniazid	InhA	Tuberculosis	Structure-based virtual screening (SBVS) and pharmacophore modeling	[22]
Pim-1 Kinase Inhibitors	Pim-1 Kinase	Cancer	Hierarchical multistage virtual screening (VS)	[23]
Epalrestat ^2^	Aldose Reductase	Diabetic neuropathy	MD and SBVS	[24]
Flurbiprofen	Cyclooxygenase-2	Rheumatoid arthritis, Osteoarthritis	Molecular docking	[25,26]
STX-0119	STAT3 ^1^	Lymphoma	SBVS	[27]
Norfloxacin	Topoisomerase II, IV	Urinary tract infection	SBVS	
Dorzolamide	Carbonic anhydrase	Glaucoma, cystoid macular edema	Fragment-based screening	[28]

^1^ Signal transducers and transcription activators (STATs). ^2^ Currently being sold in Japan under the brand name Kinedak®.

## Abbreviations

AI	Artificial intelligence
DL	Deep learning
HTS	High throughput screening
vdW	van der Waals
VS	Virtual screening
SBVS	Structure-based virtual screening
HIV	Human Immunodeficiency Virus
GA	Genetic algorithm
MC	Monte Carlo
SVM	Support vector machine
VAE	Variational autoencoder
RF	Random forest
ANN	Artificial neural network
DNN	Deep neural network
GAN	Generative adversarial network
ADMET	Absorption, distribution, metabolism, excretion and toxicity
GSK	GlaxoSmithKline
RANC	Reinforced adversarial neural computer
RL	Reinforcement learning
MD	Molecular dynamics
GPCRs	G-protein-coupled receptors
STATs	Signal transducers and transcription activators
3D	Three-dimensional
RNN	Recurrent neural network
ML	Machine learning
SBDD	Structure-based drug design
PDB	Protein data bank
NN	Neural Network
QSAR	Quantitative structure–activity relationship
QED	Quantitative estimate of drug-likeness
SMILES	Simplified molecular-input line-entry system
